# Temporal Dynamics of the Scale for the Assessment and Rating of Ataxia in Spinocerebellar Ataxias

**DOI:** 10.1002/mds.29255

**Published:** 2022-10-23

**Authors:** Paul Moulaire, Pierre Emmanuel Poulet, Emilien Petit, Thomas Klockgether, Alexandra Durr, Tetsuo Ashisawa, Sophie Tezenas du Montcel, for the READISCA Consortium

**Affiliations:** ^1^ Sorbonne Université, Paris Brain Institute, INSERM, Institut Pierre Louis d'Épidémiologie et de Santé Publique, INRIA, CNRS APHP Paris 75013 France; ^2^ Sorbonne Université Paris Brain Institute, INSERM, INRIA, CNRS, APHP Paris France; ^3^ German Center for Neurodegenerative Diseases (DZNE) Bonn Germany; ^4^ Weill Cornell Medicine at the Houston Methodist Research Institute Houston Texas USA

**Keywords:** spinocerebellar ataxia, Scale for the Assessment and Rating of Ataxia, disease course mapping

## Abstract

**Background:**

The Scale for the Assessment and Rating of Ataxia (SARA) is the reference clinical scale to assess the severity of cerebellar ataxia. In the context of upcoming therapeutic trials, a reliable clinical outcome is needed to assess the efficiency of treatments.

**Objective:**

The aim is to precisely assess and compare temporal dynamics of SARA and a new f‐SARA.

**Methods:**

We analyzed data from four cohorts (EUROSCA, RISCA, CRC‐SCA, and SPATAX) comprising 1210 participants and 4092 visits. The linearity of the progression and the variability were assessed using an ordinal Bayesian mixed‐effect model (Leaspy). We performed sample size calculations for therapeutic trials with different scenarios to improve the responsiveness of the scale.

**Results:**

Seven of the eight different items had a nonlinear progression. The speed of progression was different between most of the items, with an average time for a one‐point increase from 3.5 years [3.4; 3.6] (median, 95% credible interval) for the fastest item to 11.4 [10.9; 12.0] years. The total SARA score had a linear progression with an average time for a one‐point increase of 0.95 [0.92; 0.98] years. After removing the four last items and rescaling all items from 0 to 4, variability increased and progression was slower and thus would require a larger sample size in a future therapeutic trial.

**Conclusion:**

Despite a heterogeneous temporal dynamics at the item level, the global progression of SARA was linear. Changing the initial scale deteriorates the responsiveness. This new information about the temporal dynamics of the scale should help design the outcome of future clinical trials. © 2022 The Authors. *Movement Disorders* published by Wiley Periodicals LLC on behalf of International Parkinson and Movement Disorder Society.

Spinocerebellar ataxias (SCA) are progressive, neurodegenerative, and heterogeneous diseases that mainly affect the cerebellum, brainstem, and spinal cord. To date, at least 50 distinct genetic SCAs have been identified. The most common are SCA1, SCA2, SCA3, and SCA6, which together affect more than half of all families with dominantly inherited ataxia and are due to translated CAG repeat expansions in the respective genes.^1^ With a global prevalence from 1 to 5 per 100,000, SCAs are rare.^2^ Temporal dynamics of onset and progression are different depending on the genetic subtype: SCA1, SCA2, and SCA3 CAG repeat‐expansion carriers typically develop ataxia in the fourth decade of life, whereas the onset of ataxia in SCA6 is about 20 years later.^3^ These diseases progressively affect the daily lives of participants, with increasing gait and speech difficulties; abnormal eye movements; and non‐ataxia symptoms, such as spasticity and parkinsonism.^4^, ^5^ The repeat‐expansion SCAs lead to premature death, and there is no known cure to date, with only a few options of symptomatic treatment with modest effects. New therapies targeting the underlying pathology are currently in development.^6^

In 2006, the Scale for the Assessment and Rating of Ataxia (SARA) was developed to assess the presence and the severity of ataxia.^7^ SARA was validated for participants with SCA, translated into several languages,^8^, ^9^ and validated in related diseases such as Friedreich ataxia.^10^ It is at present the reference scale for the clinical evaluation of ataxia. Most of the studies assessed the global progression of ataxia throughout the SARA sum score, and some recent study also focused on the item progression but with a small sample size (between 20 and 190) and with models that did not allow to precisely assess the linearity of progression.^11^, ^12^, ^13^, ^14^ In the present study, we applied an ordinal mixed‐effect model to analyze longitudinally acquired data of SCA1, SCA2, SCA3, and SCA6 expansion carriers from four international cohorts (EUROSCA, RISCA, CRC‐SCA, and SPATAX).

We wanted to compare and validate SARA as a clinical outcome measure. Our aims were to (1) assess the progression at the item level, (2) compare the mean progression of the different items, and (3) assess the global progression of the SARA sum score and of a new version of SARA (f‐SARA), recently used as a primary endpoint in a therapeutic trial^15^ conducted by Biohaven.

## Patients and Methods

### Participants

Data from four existing cohorts of SCA mutation were pooled together: three cohorts with affected participants (EUROSCA,^16^ CRC‐SCA,^17^ and SPATAX^18^) and one cohort of pre‐ataxic carriers (RISCA^19^). From these cohorts that included participants with different SCA types, we selected the participants with a pathological CAG expansion in *ATXN1*, *2*, *3* and *CACNA1A*.

The EUROSCA study was conducted at 17 European centers. Participants were eligible when they had progressive, otherwise‐unexplained ataxia, and a pathological CAG expansion in *ATXN1, 2, 3* and *CACNA1A*. Participants were consecutively recruited between July 2005 and August 2006. Participants were seen at a baseline visit, followed by annual visits for 3 years. After the initial 3‐year observation period, study participants entered an extension phase in which study assessments were recorded in connection with routine visits resulting in irregular intervals between the visits.^16^

The CRC‐SCA study was conducted at 12 U.S. centers. Participants were eligible if they had a pathological CAG expansion in *ATXN1*, *2*, *3* and *CACNA1A* and were aged at least 6 years. Participants with concomitant disorders that affected SARA and other ataxia measures used in the study were excluded. The study started in April 2010. The clinical evaluation was performed at the baseline visit and every 6 months thereafter until 2 years from the baseline visit or until the end of August 2012.^17^

The RISCA study was conducted at 14 European centers. To be eligible, individuals had to have no ataxia and be aged 18 to 50 years if directly related to individuals with SCA1, SCA2, or SCA3 or 35 to 70 years if directly related to individuals with SCA6. The study started in September 2008 and ended in December 2011, and participants were seen every 18 months.^19^ We selected from the database participants with a positive genetic test.

The SPATAX cohort was coordinated at the Pitié‐Salpêtrière University Hospital in France and hosted at the Paris Brain Institute. Participants were eligible when they had a pathological CAG expansion in *ATXN1*, *2*, *3* and *CACNA1A* and at least one available SARA score. The inclusion started in 2005 (first visit with SARA assessed) until 2020, and the average baseline‐ to follow‐up interval was 1 year.^18^

### Outcomes

To assess the progression of ataxia, we used SARA, which comprises eight items. The SARA sum score ranges from 0 to 40, with zero indicating the absence of ataxia and 40 being the most severe degree of ataxia.^7^ Items 1 to 3 assess posture and gait: gait (maximum of 8), stance (maximum of 6), and sitting (maximum of 4). Item 4 (maximum of 6) assesses communication. Items 5 to 8 assess the kinetic function of the upper and lower limbs^20^: finger‐chase, finger‐nose test, fast alternating hand movements, and heel‐shin slide (each maximum of 4).

A new scale, m‐SARA, was originally devised by Biohaven as a shortened retrospective version of SARA. m‐SARA consisted of items 1 to 4, each with a score range of 0 to 4. Scores were retrospectively derived from SARA by combining one or several score categories into a single one according to predefined rules. As the FDA did not accept m‐SARA, the new f‐SARA was created with input from the FDA and used as an outcome in Biohaven troriluzole trials.^15^ Like m‐SARA, f‐SARA consists of items 1 to 4, each with a score range of 0 to 4, but with minor changes in the score categories. f‐SARA is supposed to be applied prospectively. Nevertheless, there remains a close relationship between SARA and f‐SARA. To compare SARA and f‐SARA in existing data sets possible, we defined a mapping algorithm (Fig. S1) that allows transformation of SARA into f‐SARA data. It must be noted that transformation of SARA data into f‐SARA is only an approximation, because f‐SARA scores were not obtained prospectively. In our study, we compared the temporal dynamics of the f‐SARA scale, obtained with the transformation of SARA, with the original SARA scale (Table 1).

**TABLE 1:** Participant characteristics according to SCA type

Variable	SCA Type				Overall, *N* = 1210^a^	*P*‐value^b^
	SCA1, *N* = 267^a^	SCA2, *N* = 331^a^	SCA3, *N* = 410^a^	SCA6, *N* = 202^a^		
Age (y)	44.0 (32.0, 54.0)	46.0 (34.0, 54.0)	48.0 (40.0, 57.0)	64.0 (55.5, 72.0)	49.0 (38.0, 60.0)	<0.001
Sex—female	132 (50%)	153 (47%)	200 (50%)	99 (49%)	584 (49%)	0.9
Age at onset (y)	38.0 (30.0, 45.2)	36.0 (26.5, 45.0)	39.0 (31.0, 47.0)	55.0 (46.0, 61.0)	40.0 (31.0, 50.0)	<0.001
Disease duration (y)	7.0 (5.0, 12.8)	10.0 (7.0, 15.0)	10.0 (5.8, 15.0)	9.0 (5.0, 15.0)	10.0 (5.0, 15.0)	<0.001
Number of visits	3.0 (2.0, 4.0)	4.0 (2.0, 5.0)	3.0 (1.0, 4.0)	4.0 (2.0, 5.0)	3.0 (2.0, 5.0)	<0.001
Follow‐up time (y)	3.8 (1.5, 6.2)	3.3 (1.8, 6.0)	2.9 (1.4, 5.7)	3.1 (1.5, 5.0)	3.1 (1.5, 5.9)	0.019
CAG repeat length	46.5 (44.0, 50.0)	39.0 (37.0, 41.0)	70.0 (67.0, 72.0)	22.0 (22.0, 22.0)	45.0 (38.0, 67.0)	
SARA score (max 40)	10.5 (4.2, 17.0)	13.0 (8.0, 18.0)	11.0 (6.5, 18.5)	13.0 (8.5, 18.5)	12.0 (6.6, 18.0)	0.006
Item 1—gait (max 8)	2.0 (1.0, 4.0)	3.0 (2.0, 4.0)	3.0 (2.0, 6.0)	3.0 (2.0, 6.0)	3.0 (2.0, 5.0)	<0.001
Item 2—stance (max 6)	2.0 (0.0, 3.0)	2.0 (1.0, 3.0)	2.0 (1.0, 3.0)	2.0 (1.0, 3.0)	2.0 (1.0, 3.0)	0.055
Item 3—sitting (max 4)	0.0 (0.0, 1.0)	0.0 (0.0, 1.0)	0.0 (0.0, 1.0)	0.0 (0.0, 1.0)	0.0 (0.0, 1.0)	<0.001
Item 4—speech (max 6)	2.0 (0.0, 3.0)	2.0 (1.0, 3.0)	1.0 (0.0, 2.0)	2.0 (1.0, 3.0)	2.0 (1.0, 3.0)	<0.001
Item 5—finger‐chase (max 4)	1.0 (0.5, 1.5)	1.0 (1.0, 2.0)	1.0 (0.5, 1.5)	1.0 (1.0, 2.0)	1.0 (1.0, 2.0)	<0.001
Item 6—finger‐nose (max 4)	1.0 (0.0, 1.5)	1.0 (1.0, 2.0)	1.0 (0.0, 1.0)	1.0 (0.5, 1.5)	1.0 (0.0, 1.5)	<0.001
Item 7—hand fast (max 4)	1.0 (0.0, 2.0)	1.5 (1.0, 2.0)	1.5 (0.5, 2.5)	1.5 (1.0, 2.5)	1.5 (0.5, 2.0)	0.002
Item 8—heel‐shin (max 4)	1.0 (0.5, 2.0)	1.5 (1.0, 2.5)	1.5 (1.0, 2.0)	1.5 (1.0, 3.0)	1.5 (1.0, 2.0)	0.002

^a^ Median (IQR); *n* (%).

^b^ Kruskal–Wallis rank‐sum test; Pearson's χ^2^ test.

### Statistical Analysis

#### Descriptive Statistics

Baseline data were summarized using frequencies and percentages for qualitative data and median and interquartile range (IQR) for quantitative variables and compared between genotypes using Pearson's χ^2^ test (assumptions were checked) for qualitative data and Kruskal–Wallis rank‐sum test for quantitative variables. For significant differences, pairwise Wilcoxon rank‐sum tests were used with adjusted Hochberg *P*‐values. Analyses were performed using R, version 4.1.2, and Python, version 3.1. All tests were two‐sided with a type I error rate of 0.05. Linear regression analysis and 95% confidence interval for Pearson's correlation coefficient were computed using the R package “stats.”

#### Model Description

To estimate SARA temporal dynamics, we used a nonlinear Bayesian mixed‐effect model,^21^ that is, model disease progression. It captures the temporal multivariate progression of outcomes and is robust to missing data.^22^ As in other mixed‐effect models, fixed effects (referred to as population parameters) and random effects per individual, assumed to follow a Gaussian distribution (referred to as individual parameters), are computed using the model. The model first determined the population parameters so that the mean value of all individual parameters is 0. To do so, it iteratively performed joint estimations of these population parameters with the individual ones to minimize errors between the idealized trajectory and the individual projections.

The resulting idealized trajectory therefore represents the typical participant in the studied population (referred to as the population progression). We used the open‐source software Leaspy, ordinal version, to estimate the model parameters from a longitudinal data set.^15^

The typical progression of the disease was characterized by the population parameters delta, corresponding to the average time (in years) spent at each level of the score. For instance, delta 1 is the average time spent at SARA score 1. The temporal dynamics of each participant was characterized by two individual parameters, one for the speed of progression (acceleration factor, Xi) and the other for the start of progression (time shift, Tau). The start of progression characterized the age in years at which the SARA score changed from zero to one, whereas Xi is the multiplicative factor compared to the population progression (Appendix S1).

#### Prior Distribution, Posterior Estimation, and Convergence Check

The prior distribution for population parameters was chosen to be noninformative, normally distributed with a mean of 0 and a standard deviation (SD) of 10^6^. The prior distribution for the individual parameters is also normally distributed, with a mean and SD computed on observed data and updated at each MCMC (Markov Chain Monte Carlo) iteration. Posterior parameter estimates were generated using an MCMC,^23^ and a stochastic approximation of expectation‐maximization algorithm^24^ was used to estimate the parameters by maximization of the likelihood.^25^ After the 5500 warm‐up samples were discarded, posterior estimates were derived from a further 3500 samples across all chains (no thinning) and were assessed for chain stability and convergence using visualization of trace plots and Geweke statistic.^26^

#### Temporal Dynamics of the SARA

The deltas were estimated using a multivariate model (eight items as a simultaneous function of the patient's age) for analysis at the item level and using a univariate model (SARA score as a function of the patient's age) for analysis of the global progression of the scale. Posterior distributions of the parameters (referred to as distributions) were used for analysis.

The linearity of the progression was assessed by inspecting the overlap between the distributions of the deltas and the computation of the median and 95% credible interval (95% CI) of the differences between the distributions of deltas. We rejected the hypothesis that the progression was linear when there was no overlap, and zero was not in the 95% CI of the difference between the distributions of deltas.

The differences in speed of progression between items were assessed by comparing their mean delta. This mean delta is the average time spent in each level, also characterizing the average time for a one‐point increase. The variability in progression was measured using the IQR of the average progression of each item.

#### Individual Parameters, Cofactor Analysis, and Internal Consistency

The difference between speed of progression (acceleration factor Xi) and age of start of progression (time shift Tau) of the disease was compared between SCA type, continent, gender, and cohort using the individual parameters estimated by the model. The relationship between age of start of progression and CAG repeat length was evaluated using Pearson's correlation test (normality assumptions were graphically assessed). Internal validity of the scale was analyzed by assessing its reliability through Cronbach's α computation (the Bootstrap confidence interval was calculated by taking 1000 samples with replacement from the data, calculating for each α, and computing the quantiles).

### Data Sharing

Anonymized data can be obtained from the coordinators of the four source studies on reasonable request.

## Results

### Baseline Characteristics of the Participants

A total of 1210 participants met our selection criteria from 4092 visits; 267 (22%) SCA1 participants were significantly younger at inclusion, aged 44 [32; 54] (median IQR) years, than SCA3 participants aged 48 [40; 57] years (*P* < 0.001) and SCA6 participants aged 64 [55; 72] years (*P* < 0.001) with a shorter disease duration: 7 [5; 12.8] years versus 9 [5; 15] years for SCA6 participants (*P* = 0.045) and 10 [7; 15] years for SCA2 and SCA3 participants (*P* < 0.001 and *P* < 0.001, respectively). These SCA1 participants had lower SARA scores at baseline than SCA2 participants (10.5 [4.2; 17] vs. 13.0 [8; 18], *P* = 0.014) and SCA6 participants (13.0 [8.5; 18.5], *P* = 0.029). As expected, SCA6 participants were older at baseline with an older age at onset. At baseline, the maximum score was reached for 6.67% of patients for items gait and stance, 3.34% for sitting, 0.79% for speech, 1.67% for finger‐chase, 1.35% for finger‐nose, 1.99% for hand fast, and 3.57% for heel‐shin.

### Different Temporal Dynamics at the Item Level

**FIG 1:**
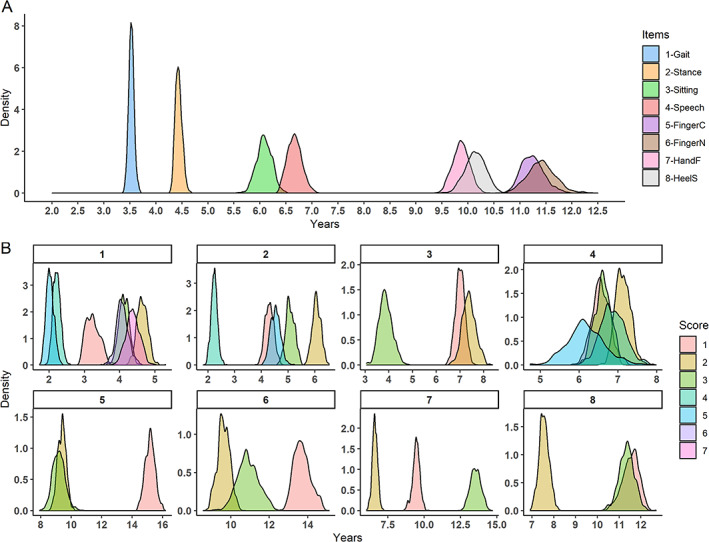
Score progression at the item level. (**A**) Posterior distribution of the average time for a one‐point increase in the corresponding item. For instance, the blue distribution is the average time for a one‐point increase in item 1, gait, of the SARA. The median and 95% CI (credible interval) of each distribution are presented in Table S1. (**B**) Each panel corresponds to the progression of one item of the SARA. In one panel, each distribution represents the time spent in each level of this item. For instance, for panel B‐1, gait, the red distribution, score1, is the average time spent at gait score 1. [Color figure can be viewed at wileyonlinelibrary.com]

There were discrepancies between the average progressions of the different items (Fig. 1A; Table S1). Items gait and stance progressed faster (average time for a one‐point increase between 2.5 and 5 years), sitting and speech had an intermediate progression rate (average time for a one‐point increase between 5 and 7.5 years), and the last four items had a slower progression (average time for a one‐point increase between 9 and 12.5 years). Items 1 to 4 had a lower variability of progression with an IQR between 0.07 and 0.19, whereas items 5 to 8 had an IQR between 0.22 and 0.34.

**FIG 2:**
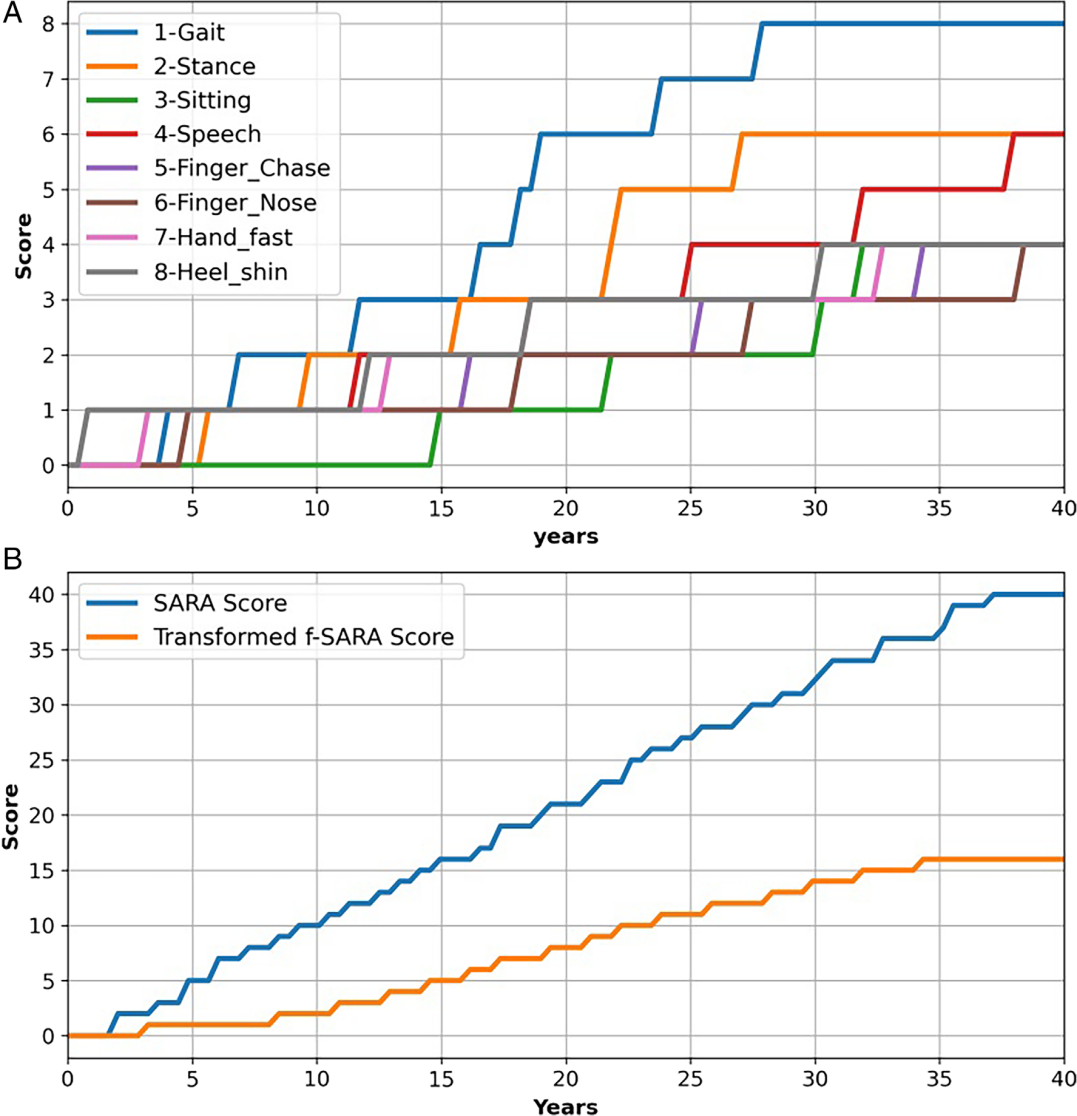
Average score progression as a function of years from start of progression. (**A**) Average trajectory of progression of each item comprising the SARA. (**B**) Average trajectory of the SARA and the comparison with the average progression of the transformed f‐SARA. The level of the score corresponds to that obtained using the maximum likelihood. [Color figure can be viewed at wileyonlinelibrary.com]

The evolution was not linear within the items, with different times for a one‐point increase for seven of the eight items (Fig. 2A). The shortest delta of the speech item was 6.14 [5.31; 7.18] (median, IQR), whereas the longest was 7.12 [6.78; 7.52], with a nonsignificant difference of 0.95 [−0.12; 1.96] (Fig. 1B). For all the other items, the progression was not linear, with significant different deltas. Item gait had a maximum difference in progression of 2.63 [2.26; 2.97] years between the fastest and the slowest delta, item stance 3.83 [3.46; 4.23], item sitting 3.50 [2.70; 4.34], item finger‐chase 5.97 [5.11; 6.91] years, item finger‐nose 4.07 [3.13; 5.09] years, item hand fast movements 6.91 [6.13; 7.67] years, and item heel‐shin test 3.99 [3.12; 4.81] years.

### Global Progression and Internal Consistency of SARA and Transformed f‐SARA

The global progression of the SARA was linear between scores 3 and 36 based on the overlap between the distribution of deltas (Fig. 2B; Fig. S2A), whereas the transformed f‐SARA was linear only between scores 3 and 10 with a floor and a ceiling effect (Fig. 2B; Fig. S2B).

With the same clinical progression, the SARA had a faster progression than f‐SARA, with an average time for a one‐point increase (delta) of 0.95 years [0.92; 0.98] versus 2.11 years [2.04; 2.18]. Moreover, with a shorter IQR of the average progression (0.019 vs. 0.041), the SARA had a lower variability in progression confirming a more regular progression.

The five deltas of the SARA further from the average progression were delta 39: 1.41 [0.96; 2.01] years; delta 36: 1.36 [1.01; 1.84]; delta 2: 1.28 [1.08; 1.46]; delta 1: 1.19 [0.99; 1.38]; and delta 38: 0.42 [0.19; 0.71]. They were at the beginning and the end of the scale, resulting in a linear progression of SARA score between 4 and 36. The five deltas of the f‐SARA further from the average progression were delta 6: 1.48 [1.34; 1.64] years; delta 5: 1.58 [1.43; 1.75]; delta 8: 1.60 [1.41; 1.78]; delta 2: 2.53 [2.33; 2.72], and delta 1: 4.94 [4.63; 5.19]. The two first deltas had the highest value and 95% CI resulting in a slower and more variable progression at the beginning of the f‐SARA.

Internal consistency of the global SARA was high, with a Cronbach's α of 0.922 (95% confidence interval: [0.918; 0.925]). Suppression of any item resulted in a decrease in the internal consistency. The f‐SARA had a lower internal consistency with a Cronbach's α of 0.898 [0.893, 0.903] (Table S2).

### Influence of Cofactors on SARA Temporal Dynamics

**FIG 3:**
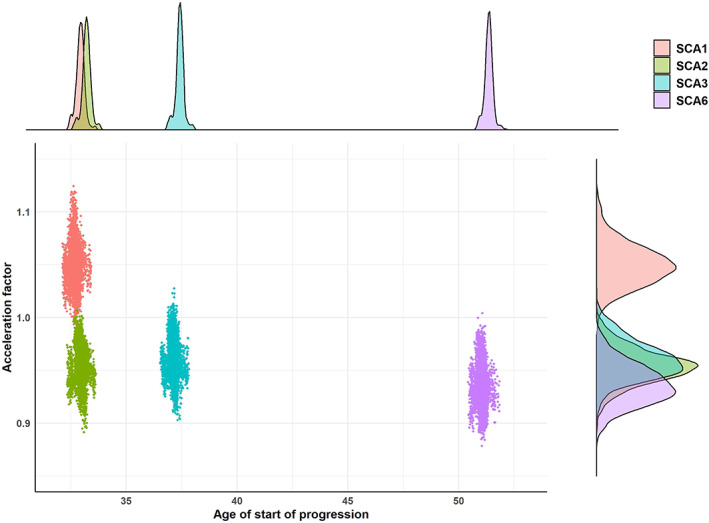
Temporal dynamics per spinocerebellar ataxia type. Each point of the central panel represents the mean acceleration factor and age of start of progression computed at each MCMC (Markov Chain Monte Carlo) iteration (after the burn‐in) per SCA type. The top panel is the posterior distribution of the average age of start of progression per SCA type, whereas the right panel is the posterior distribution of the average acceleration factor per SCA type. [Color figure can be viewed at wileyonlinelibrary.com]

SCA1 individuals had the fastest average speed progression of the disease with an acceleration factor (Xi) of 1.05 [1.01; 1.09], whereas SCA2 participants had 0.96 [0.92; 0.99], SCA3 participants 0.96 [0.93; 0.99], and SCA6 participants 0.94 [0.90; 0.98] (Fig. 2). In addition, SCA1 and SCA2 individuals had the lowest estimated age of start of progression with 32.7 [32.2; 33.1] and 33.0 [32.5; 33.4] years, respectively, followed by SCA3 participants with 37.2 [36.7; 37.6] years and SCA6 participants with 51.0 [50.6; 51.4] years (Fig. 3).

Participants from European cohorts (EUROSCA, RISCA, and SPATAX) had a lower age of start of progression of the disease and a faster speed of progression (Fig. S4A). At the cohort level, CRC and SPATAX participants had the same temporal dynamics, whereas EUROSCA and RISCA participants had a faster speed of progression and earlier age of start of the disease (Fig. S4B). There was no effect of gender in the study (Fig. S5).

The estimated age of start of progression was correlated with the logarithm of the CAG repeat length particularly for SCA1, SCA2, and SCA3 participants (SCA1: *r* = −0.71 [−0.77, −0.64] (*P* < 0.001); SCA2: *r* = 0.74 [−0.79, −0.68] (*P* < 0.001); SCA3: *r* = 0.69 [−0.74, −0.63] (*P* < 0.001); and SCA6: *r* = −0.33 [−0.45, −0.20] (*P* = 0.001)) (Fig. S3).

### Sample Size Calculation for Therapeutic Trials

**FIG 4:**
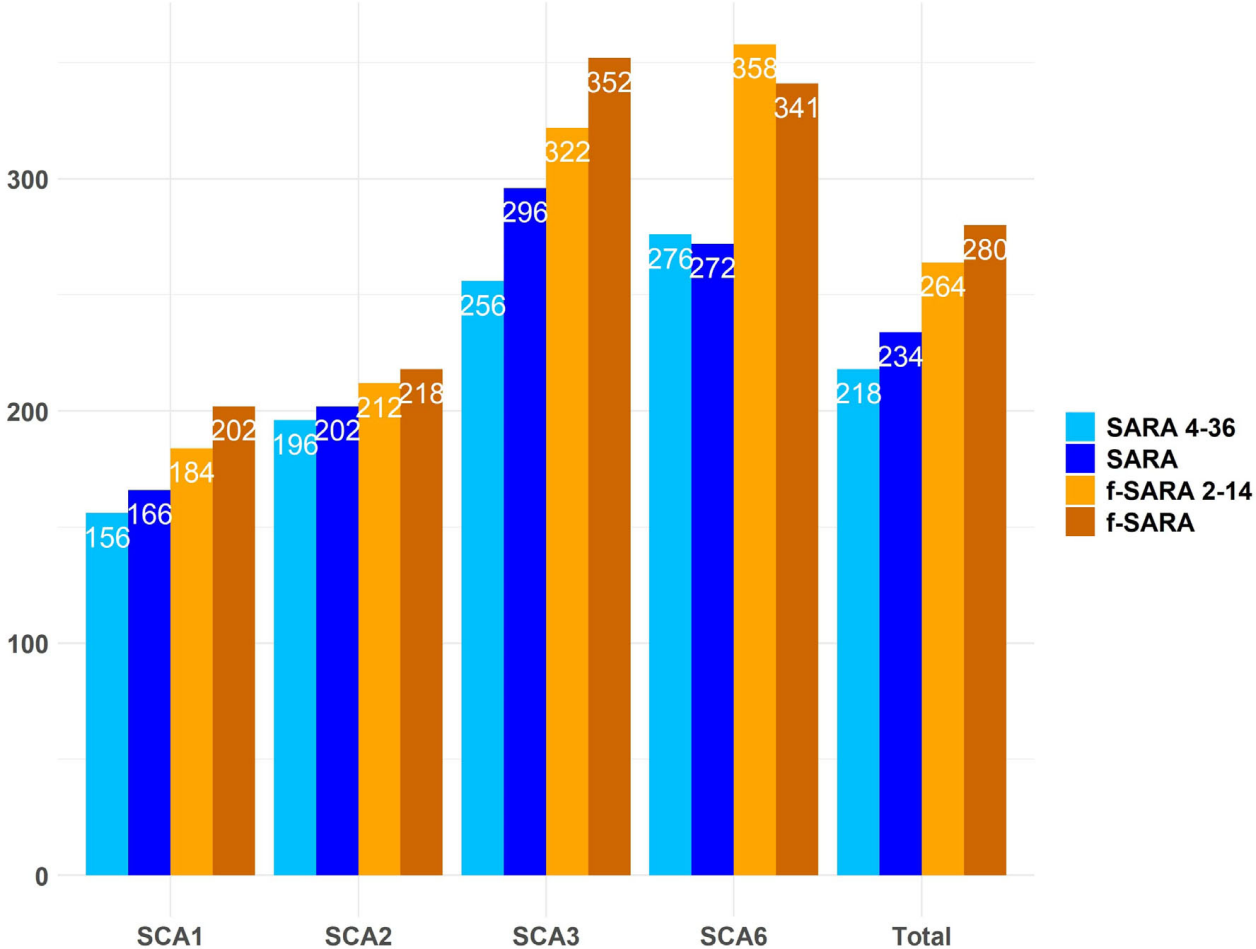
Total number of participants required for a therapeutic trial. Sample size for a two‐group interventional trial (1:1 ratio per group) of 12‐month duration with a treatment effect of 50% on the disease progression, a power of 90%, and an α of 5%. SCA, spinocerebellar ataxia; total, heterogeneous group of SCA; f‐SARA, transformed scale; SARA 4 to 36, inclusion criteria: participants between SARA scores 4 and 36. [Color figure can be viewed at wileyonlinelibrary.com]

Based on the estimated disease course of the SARA score, we computed the sample size for two equal‐sized groups with an interventional trial of 12 months and a mean evolution in the placebo group of 1.08 (SD = 1.27), estimated without the patients from the RISCA cohort, and with a treatment effect of 50% on disease progression according to Jacobi et al^27^ (power of 90%, two‐sided α of 5%). Groups with SCA1 participants needed fewer subjects because the progression speed of the disease was faster, whereas groups with SCA3 participants needed more subjects because the progression was slower and had more variability (Table S3). The transformation of SARA into f‐SARA decreased the speed and linearity of progression and increased the variance leading to an increase in the required sample size. Selecting participants with baseline SARA between scores 4 and 36 reduced the required sample size except in the case of a trial with only SCA6 participants. A scenario with f‐SARA between scores 2 and 14 reduced the sample size required but remained bigger than with original SARA (Fig. 4). With a heterogeneous group of SCA, 280 participants are needed with the transformed f‐SARA, 234 with the original SARA, and 218 if the inclusion criteria include a SARA score between 4 and 36.

## Discussion

In this study, we show the temporal dynamics of the SARA and the impact of its modification on responsiveness to change. At the item level, we found a heterogeneous temporal dynamics, qualitatively with a nonlinear progression for seven of eight items and quantitatively items progressing significantly faster than others do. Given the differences in the number of scoring options between the items, it is expected that progression at gait (eight points) and stance (six points) is faster than that of the last four items (four points each). Despite these discrepancies, the global SARA had a linear and stable progression. This important result suggests that all items are complementary and that they each provide specific information at different stages of the disease. The most stable and linear temporal dynamics was observed for SARA between 3 and 36, making this interval the best to be used as inclusion criteria, over the threshold of three defined in the literature.^19^ Linearity of the global scale is a very useful information for the design of therapeutic trials, particularly in rare diseases. Indeed, it allows including participants at different stages of the disease although expecting the same natural progression.

Both univariate and multivariate detailed analyses of temporal dynamics have been made possible due to a disease course‐mapping model. This model is particularly useful for modeling several outcomes simultaneously although being robust to missing data.^19^, ^29^ The ordinal version is extremely well suited to the detailed study and modeling of the evolution of a score, without making any assumptions about the overall shape of the progression. This model has already been used in other progressive diseases such as Parkinson's disease and Alzheimer's disease.^28^, ^29^, ^30^, ^31^ The results about the influence of genetic factors on the temporal dynamics of the SARA score were already known. Correlation between pathological CAG repeats and age at onset was precisely studied and reported.^32^ In our study, the age of start of progression estimated by the model was between 32.7 and 37.2 years for SCA1, SCA2, and SCA3 participants and 51.0 years for SCA6 participants. These estimations are close to those described in the literature, with age 36.8 to 40.4 years for SCA1, SCA2, and SCA3 participants and 52.2 years for SCA6 participants.^12^ The concordance between our results and those reported in the literature contributes to validate the use of this model and to reinforce the new findings.

At first glance, the modifications of the SARA to the f‐SARA by removing the four appendicular items and rescaling all others out of four appear logical. Indeed, we noticed in the analysis at the item level that the four items removed had not only slower speed progression but also larger IQR involving more variability. However, these items still provide information on the progression of the disease, and removing them worsens the performance of the new scale by decreasing granularity, increasing variability, and slowing the rate of progression. This information is confirmed by the study of internal consistency, which is maximal only by keeping all items and by the sample size calculation that requires fewer individuals using the original SARA. A recent clinical trial using the prospective f‐SARA did not reach statistical significance in the overall SCA population.^33^ Although the f‐SARA transformed in our study and f‐SARA used by Biohaven are different, it is likely that their evolutions are similar. Therefore, the f‐SARA is more variable and progresses slower than the classical SARA. With a mean baseline f‐SARA score of 4.9 and a mean score of 5.2 at 48 weeks, the placebo group progressed at a pace of only 0.3 points in almost 1 year, tending to confirm the slow progression of f‐SARA.

Our study presents some limitations. Given the different origins of cohorts as well as the natural variability of disease progression, our data were heterogeneous, and the average model should be interpreted with caution. The bivariate analyses may be subject to confounding. As in all mixed‐effects models, the population progression is only a theoretical trajectory and should be interpreted only by considering the individual parameters of the participants. Even if the progression of the SARA in a mixed group of SCA is linear,^7^, ^11^ it is possible that the individual progression at the SCA level is not linear.^12^, ^34^ Due to limited sample size, our model could not be fitted on each SCA type independently. Even if our model was truly discriminant in terms of speed progression of the SARA between the different SCA types, we did not observe the same magnitude as described in the literature. SCA1 participants progressed 2.64 times faster than SCA6 participants in the EUROSCA study (2.11 points per year vs. 0.8 point per year),^16^ 1.87 times faster in the CRC study (1.61 vs. 0.86),^17^ and 1.24 times faster in a third study (1.23 vs. 0.99),^11^ whereas the progression was just 1.12 times faster in our study (1.11 vs. 0.99). In addition, our study and the sample size calculation were mainly focused on the SARA progression. In a therapeutic trial with pre‐ataxic participants, the primary outcome should not be the variation in SARA score but the time until conversion.^27^

Even with items rated on different maximum points and heterogeneous progressions, the global SARA had a linear and stable temporal dynamics. Moreover, SARA remains the reference to assess the severity of cerebellar ataxia, it is translated into many languages and used globally, and it is well mastered by practitioners. Other tools like SARA training tool and SARA Home exist to be learned and used.^35^, ^36^ Any modification should be done with caution to retain the good properties of the scale. As SCAs are rare, it is essential to have a common measurement scale to compare and share results and maintain a close collaboration among different countries to ensure the rapid development of a treatment.

## Author Roles

P.M., P.E.P., and S.T.M. were involved in methodology, formal analysis, investigation, data curation, writing of the original draft, reviewing, and editing of the manuscript, designing of tables and graphs, and verifying the underlying data. E.P., T.K., A.D., and T.A. were involved in data provision and reviewing and editing of the manuscript. All authors accept responsibility for the decision to submit for publication.

## Full Financial Disclosures For The Previous 12 Months

S.T.M. receives research support from Biogen. T.A. received grants from NAF and Biogen and participates in Biohaven clinical trials NCT03952806 and NCT03701399.

## Supporting information


**S1.** Supporting Information

## Data Availability

Data available on request from the authors
