# Supplementary material for: Temporal Dynamics of the Scale for the Assessment and Rating of Ataxia in Spinocerebellar Ataxias
Source: Mov Disord. 2022 Oct 23;38(1):35–44. doi: 10.1002/mds.29255 (PMC9851985; doi:10.1002/mds.29255)
Supplement: Supplementary file 1 — S1. Supporting Information [file MDS-38-35-s001.docx]

**Supplementary Table 1: Average time in years for a one-point increase per item**

| Item of the SARA scale | Median | (L, U)^1^ |
| --- | --- | --- |
| 1-Gait | 3.53 | (3.43, 3.63) |
| 2-Stance | 4.43 | (4.33, 4.57) |
| 3-Sitting | 6.08 | (5.80, 6.36) |
| 4-Speech | 6.66 | (6.42, 6.96) |
| 5-Finger_chase | 11.23 | (10.86, 11.71) |
| 6-Finger_nose | 11.41 | (10.90, 11.97) |
| 7-Hand_fast | 9.87 | (9.56, 10.18) |
| 8-Heel_shin | 10.14 | (9.76, 10.51) |

^1^L: 95% Lower credible interval, U: 95% Upper credible interval

**Supplementary Table 2, Cronbach's Alpha for SARA and f-SARA**

|  | SARA scale | | Transformed f-SARA scale | |
| --- | --- | --- | --- | --- |
| Item removed | Alpha | (L, U)1 | Alpha | (L, U)1 |
| None | 0.922 | (0.918, 0.925) | 0.898 | (0.893, 0.903) |
| 1-Gait | 0.920 | (0.916, 0.923) | 0.855 | (0.847, 0.863) |
| 2-Stance | 0.900 | (0.895, 0.904) | 0.847 | (0.838, 0.855) |
| 3-Sitting | 0.914 | (0.910, 0.917) | 0.887 | (0.881, 0.893) |
| 4-Speech | 0.906 | (0.902, 0.910) | 0.878 | (0.871, 0.884) |
| 5-Finger_chase | 0.914 | (0.911, 0.917) | NA | NA |
| 6-Finger_nose | 0.917 | (0.914, 0.920) | NA | NA |
| 7-Hand_fast | 0.909 | (0.905, 0.913) | NA | NA |
| 8-Heel_shin | 0.912 | (0.909, 0.916) | NA | NA |

^1^L: 95% Lower confidence interval, U: 95% Upper confidence interval

NA: not assessed as the item is not included in the f-SARA

**Supplementary Table 3, Treatment effect and annual variance of progression of the scores estimated by the model.**

|  | SARA 4-36 | | SARA | | f-SARA | |
| --- | --- | --- | --- | --- | --- | --- |
|  | Treatment effect | SD | Treatment effect | SD | Treatment effect | SD |
| SCA1 | 0.71 | 1.36 | 0.68 | 1.35 | 0.45 | 0.99 |
| SCA2 | 0.54 | 1.7 | 0.53 | 1.61 | 0.33 | 0.75 |
| SCA3 | 0.5 | 1.22 | 0.49 | 1.31 | 0.33 | 0.94 |
| SCA6 | 0.49 | 1.26 | 0.48 | 1.24 | 0.26 | 0.84 |
| Total | 0.55 | 1.25 | 0.54 | 1.27 | 0.35 | 0.89 |

Treatment effect: Difference of annual progression assuming a treatment effect of 50%

SD: Standard deviation of progression of the score estimated by the model for a 12 month progression.

f-SARA**=** Transformed SARA scale

SARA 4-36= Inclusion criteria: participants between SARA score 4 and 36.

**Supplementary Figure 1: Algorithm for mapping SARA onto transformed f-SARA**


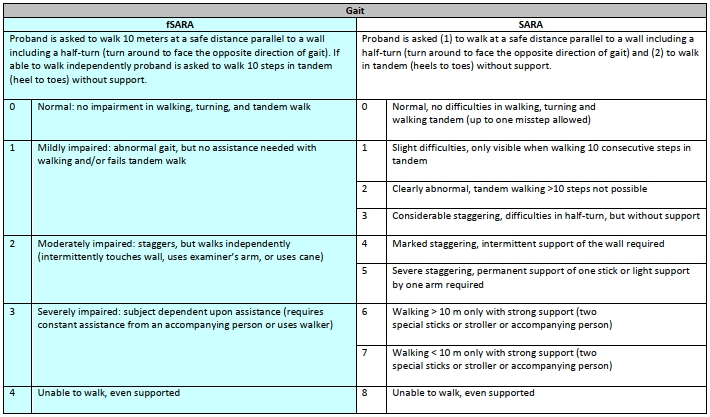

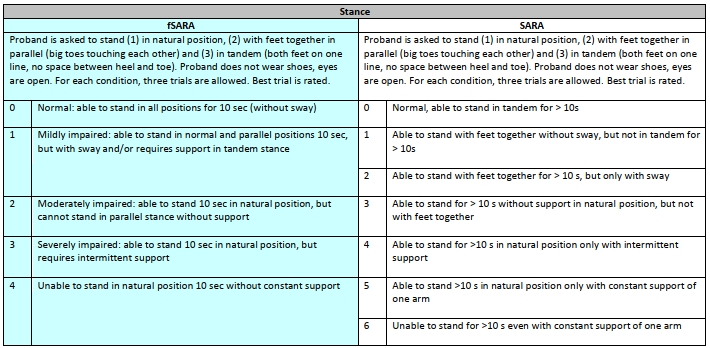

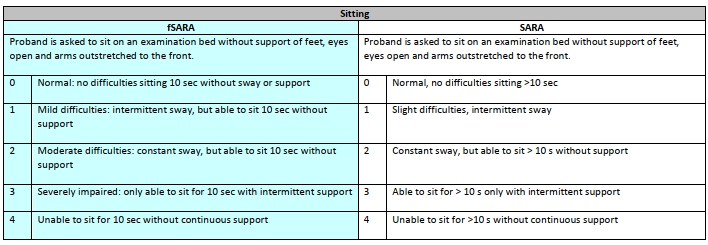

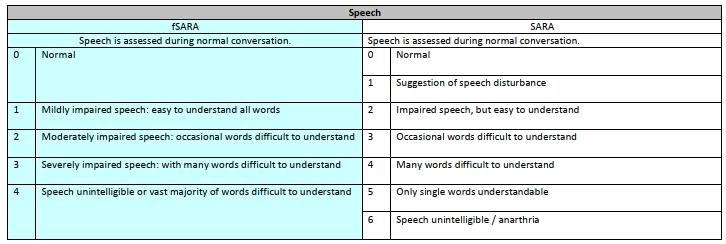


**Supplementary Figure 2: Global temporal dynamic of the SARA (A) and transformed f-SARA (B)**


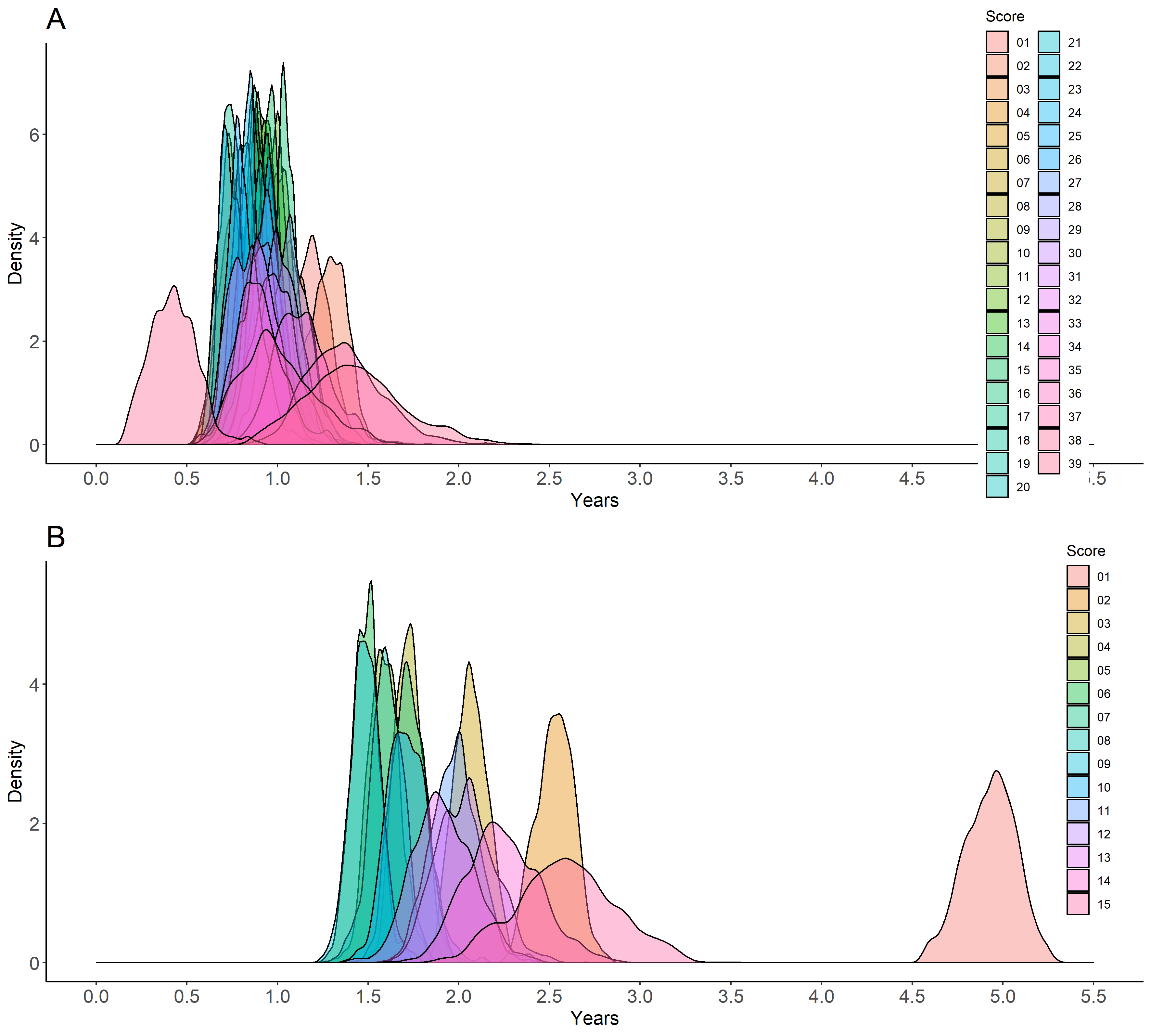


**A:** Posterior distribution of the time spent in each level of the SARA scale.

**B:** Posterior distribution of the time spent in each level of the f-SARA scale.

For instance, the red distribution, score 01 represents the time in years spent at SARA score 1 (between 4.5 and 5.3 years).

**Supplementary Figure 3: Estimated age at onset as a function of Polyglutamine-coding (CAG)n repeat expansions**


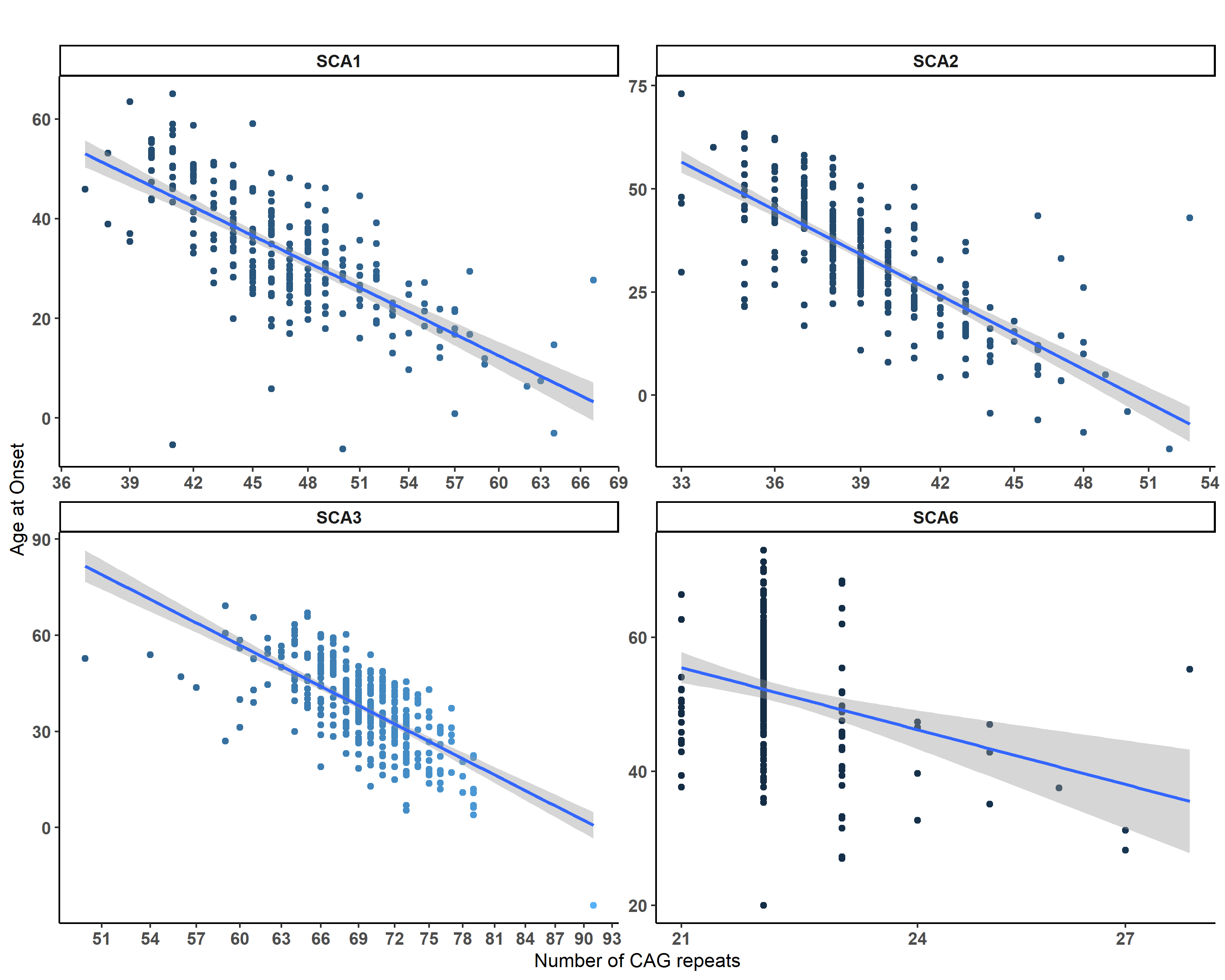
Blue lines: Fitted linear regression lines with 95% confidence bands

The X axis is in logarithm scale.

**Supplementary Figure 4: Temporal dynamic per Spinocerebellar cohort and continent**

**
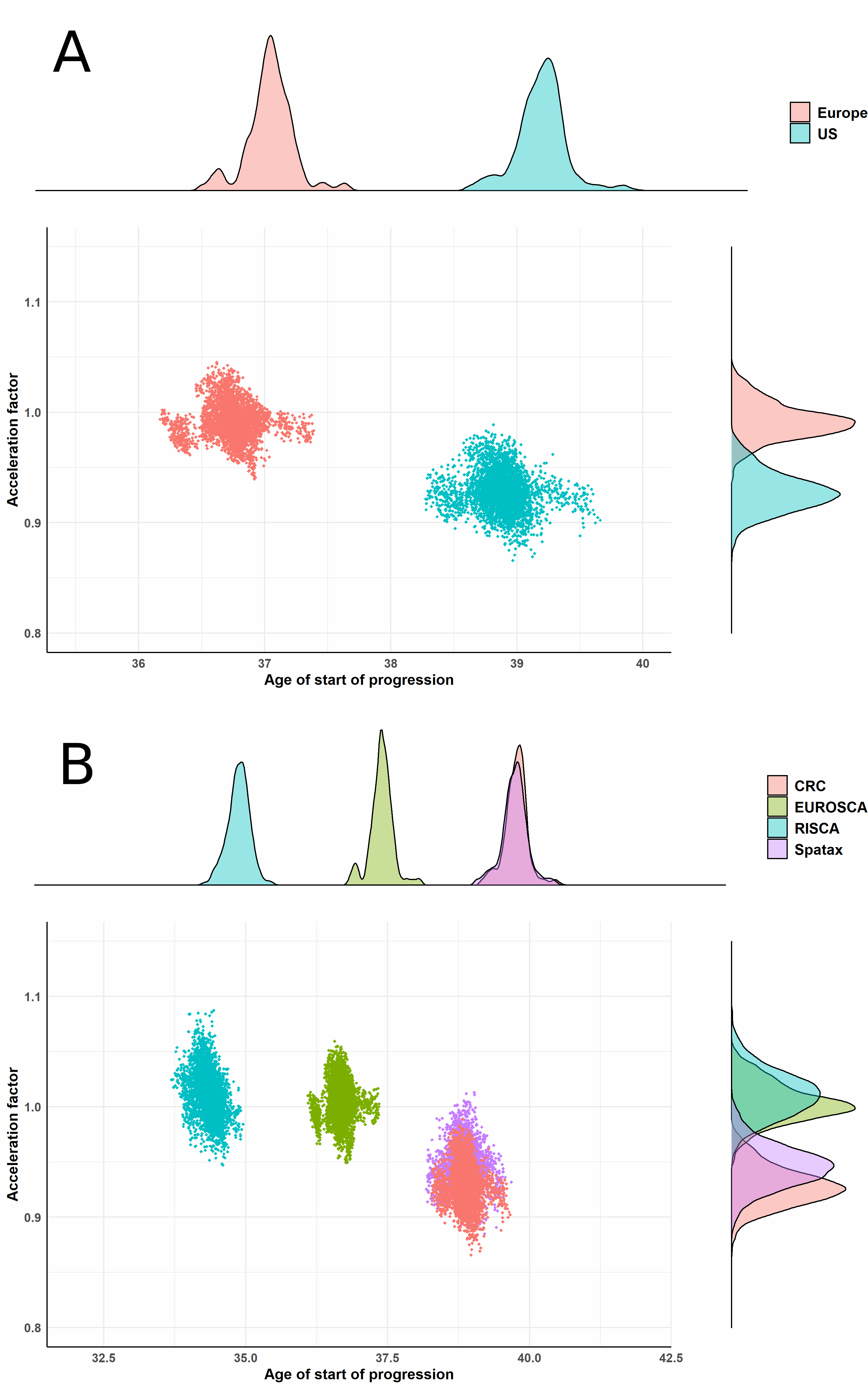
**

Each point of the central panel represents the mean acceleration factor and age of start of progression computed at each MCMC iteration (after the burn-in) per continent (A) or per cohort (B) with the corresponding distribution on the top and the right part.

**Supplementary Figure 5: Temporal dynamic per Spinocerebellar cohort and continent**

**
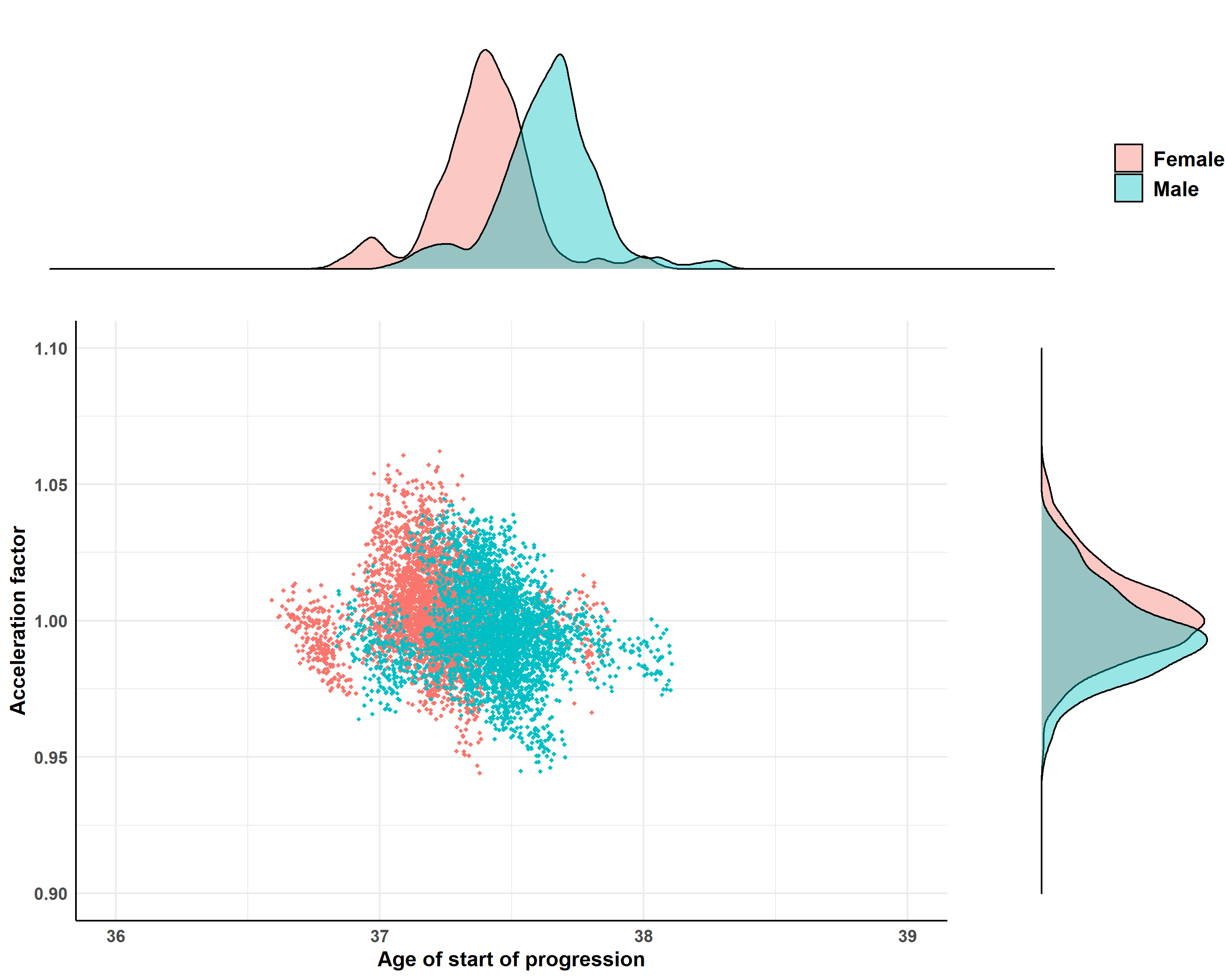
**

Each point of the central panel represents the mean acceleration factor and age of start of progression computed at each MCMC iteration (after the burn-in) per gender with the corresponding distribution on the top and the right part.

**Supplementary material-1: Mathematical description of the model:**

We provide here a mathematical description of the disease course mapping model which was first introduced in [1]. We introduce the notations and essential equations for the rest of the article. In this following method we focus on the particular case where the model takes the form of logistic curves. For a more generic approach, please refer to the original paper [1].

We consider a longitudinal dataset $\left( y_{ijk} \right)_{1\leq i\leq n,1\leq j\leq N_{i},1\leq k\leq d}$with i being the patient index, j the visit index (visit j occurs at $t_{ij}$) and k being the feature index. There are n total patients with each patient having a certain number $N_{i}$ of visits, where up to d markers are recorded. The model will be able to cope with missing data.

The geometrical model assumes that the d-dimensional vector of observations $y_{ij}$ belongs to a Riemannian manifold $M$. Each patient has thus a continuous trajectory in this manifold, and together they form a bundle of trajectories. We parametrize this non-linear longitudinal model with a mixed-effect structure. The fixed effects will represent an “average” trajectory in the manifold, which we assume is a geodesic (thus easy to parametrize: we simply need a point and a tangent vector to define such a trajectory). The random effects will account for variability in the trajectories on the manifold, but also for variability in the speed of progression along those trajectories.

Concretely the fixed effects to describe the average population trajectory, denoted $\gamma_{0}$, are the initial point $p=\gamma_{0}\left( t_{0} \right)$, and the first order derivative $v=\gamma_{0}\left( t_{0} \right)$. The random effects are decomposed into two parts: the spatial effects correspond to a shift in the manifold, which is parametrized by a space-shift $w_{i}$, and a temporal part with an individual time-reparametrization $\psi_{i}\left( t \right)=\alpha_{i}\left( t-\tau_{i} \right)$ with $\alpha_{i}$ being the acceleration factor (or more commonly $\xi_{i}=log\alpha_{i}$ being the log-acceleration) and $\tau_{i}$ being the time-shift. This time-reparametrization captures two phenomena: the possibility for a patient to be a fast/slow progressor and the potential delay of disease onset compared to the mean of the population.

We use the logistic curves instantiation of this model f which has the following form:

$$f\left( t_{ij},\xi_{i},\tau_{i},w_{i} \right)=\frac{1}{1+\left( \frac{1}{p_{k}}-1 \right)exp\left( \frac{-v_{k}e^{\xi_{i}}\left( t-\tau_{i} \right)+w_{ik}}{p_{k}\left( 1-p_{k} \right)} \right)}$$

The space-shifts $w_{i}$have the same dimension as the observations. However, in an attempt to reduce the number of parameters of the model and in order to increase the interpretability the model uses an ICA decomposition with $N_{s}$ independent sources $\left( s_{ik} \right)_{1\leq k\leq N_{s}}.$The link between the sources and the space-shifts is thus $w_{i}=As_{i}$ and for identifiability issues the columns of the mixing matrix $A$ are orthogonal to $v$**.**

In the hierarchical statistical model, the population and individual parameters are latent, and follow Gaussian prior distributions: $\xi_{i} N\left( 0,\sigma_{\xi}^{2} \right)$, $\tau_{i} N\left( \overline{\tau},\sigma_{\tau}^{2} \right)$, $s_{ik} N\left( 0,1 \right)$, $g_{k} N\left( \overline{g},\sigma_{g}^{2} \right)$ where $g_{k}=\frac{1}{p_{k}}-1$, $v_{k} N\left( \overline{v},\sigma_{v}^{2} \right)$. All the new parameters ($\sigma_{\xi},\sigma_{\tau},\sigma_{g},\sigma_{v},\overline{\tau},\overline{g},\overline{v}$) introduced here will be the model parameters.

For the ordinal model, we assume the observations lie on a discontinuous space with ordered values such as for a cognitive score. The possible values are between 0 and L. We now specify the logistic curves model for ordinal data in a way similar to a cumulative probit model from item response theory:

$$P\left( y_{ijk}\geq l \right)=\frac{1}{1+\left( \frac{1}{p_{k}}-1 \right)exp\left( \frac{-v_{k}e^{\xi_{i}}\left( t-\tau_{i}-\sum_{m=1}^{l} \delta_{k}^{m} \right)+w_{ik}}{p_{k}\left( 1-p_{k} \right)} \right)}$$

With $\delta_{m}^{k}>0$ being the expected time spent at score m for feature k. The $\delta_{m}^{k}$ are latent parameters with a log-normal distribution.

The estimation of the parameters of the model is performed with the Monte Carlo Markov chain stochastic approximation variant of the Expectation Maximization algorithm (MCMC-SAEM). The convergence of the MCMC-SAEM has been proven for distributions of the curved exponential family. The algorithm is an iterative scheme alternating between expectation and maximization steps:

- E-step: latent parameters are estimated by a Metropolis-Hastings within Gibbs sampler algorithm. This allows to compute the expectation of the log-likelihood using the sampled values of the latent parameters.
- M-step: the model parameters are updated by maximizing the expectation of the log-likelihood which is computed in closed form.

[1] J.-B. Schiratti, S. Allassonnière, O. Colliot, et S. Durrleman, « A Bayesian mixed-effects model to learn trajectories of changes from repeated manifold-valued observations », J. Mach. Learn. Res., vol. 18, nᵒ 1, p. 4840‑4872, janv. 2017.
